# Examination of BDNF Treatment on BACE1 Activity and Acute Exercise on Brain BDNF Signaling

**DOI:** 10.3389/fncel.2021.665867

**Published:** 2021-05-04

**Authors:** Bradley J. Baranowski, Grant C. Hayward, Daniel M. Marko, Rebecca E. K. MacPherson

**Affiliations:** ^1^Department of Health Sciences, Brock University, St. Catharines, ON, Canada; ^2^Centre for Neuroscience, Brock University, St. Catharines, ON, Canada

**Keywords:** Alzheimer’s disease, BDNF, exercise, beta-secretase, obesity

## Abstract

Perturbations in metabolism results in the accumulation of beta-amyloid peptides, which is a pathological feature of Alzheimer’s disease. Beta-site amyloid precursor protein cleaving enzyme 1 (BACE1) is the rate limiting enzyme responsible for beta-amyloid production. Obesogenic diets increase BACE1 while exercise reduces BACE1 activity, although the mechanisms are unknown. Brain-derived neurotropic factor (BDNF) is an exercise inducible neurotrophic factor, however, it is unknown if BDNF is related to the effects of exercise on BACE1. The purpose of this study was to determine the direct effect of BDNF on BACE1 activity and to examine neuronal pathways induced by exercise. C57BL/6J male mice were assigned to either a low (*n* = 36) or high fat diet (*n* = 36) for 10 weeks. To determine the direct effect of BDNF on BACE1, a subset of mice (low fat diet = 12 and high fat diet *n* = 12) were used for an explant experiment where the brain tissue was directly treated with BDNF (100 ng/ml) for 30 min. To examine neuronal pathways activated with exercise, mice remained sedentary (*n* = 12) or underwent an acute bout of treadmill running at 15 m/min with a 5% incline for 120 min (*n* = 12). The prefrontal cortex and hippocampus were collected 2-h post-exercise. Direct treatment with BDNF resulted in reductions in BACE1 activity in the prefrontal cortex (*p* < 0.05), but not the hippocampus. The high fat diet reduced BDNF content in the hippocampus; however, the acute bout of exercise increased BDNF in the prefrontal cortex (*p* < 0.05). These novel findings demonstrate the region specific differences in exercise induced BDNF in lean and obese mice and show that BDNF can reduce BACE1 activity, independent of other exercise-induced alterations. This work demonstrates a previously unknown link between BDNF and BACE1 regulation.

## Introduction

Clinical and epidemiological evidence highlights the fact that lifestyle factors, such as diet and exercise, are crucial in reducing the risk of late onset Alzheimer’s disease (de la Monte and Wands, 2008; Pasinetti and Eberstein, 2008; Vagelatos and Eslick, 2013; Baranowski et al., 2018b, 2020). The accumulation of amyloid beta (Aβ) plaques is a pathological hallmark of Alzheimer’s disease (Behl and Holsboer, 1998). BACE1 is the rate-limiting enzyme in the amyloidogenic pathway, resulting in the production of Aβ peptides. BACE1 has therefore been the target of several pharmaceuticals inhibitors – however, the majority of these targeted drugs have failed in clinical trials (Das and Yan, 2019). Given the lack of appropriate pharmacological regulation of BACE1, research must focus on understanding the physiological regulation of BACE1 and how diet and exercise may be utilized to prevent Alzheimer’s disease.

Epidemiological studies have identified physical inactivity and obesity as significant risk factors for Alzheimer’s disease mortality rates and disease progression (Profenno et al., 2010; Baranowski et al., 2018b). In humans, there appears to be a relationship between obesity and circulating plasma Aβ (Mayeux et al., 2003; Balakrishnan et al., 2005; Luciano et al., 2015), along with evidence to support that higher levels of plasma Aβ can be used to predict the risk of developing Alzheimer’s disease and cognitive impairments (Ida et al., 1996; Mayeux et al., 2003; Mayeux and Schupf, 2011). Further, obese rodent models display elevations in brain BACE1 expression, protein content, and activity (Thirumangalakudi et al., 2008; Zhang et al., 2009; Wang et al., 2013; MacPherson et al., 2015). On the other hand, chronic physical activity/exercise has proven to reduce Aβ levels and associated memory deficits (Adlard et al., 2005; Maesako et al., 2012, 2013). In support of a direct effect of exercise on the brain, recent work from our lab examined whether an acute bout of exercise could reduce BACE1 activity and/or content after 7 weeks of high fat feeding, similar to that seen with long-term exercise training. These studies revealed that a single bout of exercise was able to reduce BACE1 content and activity in the brains of obese mice (MacPherson et al., 2015; Yang A. J. et al., 2019); however, the exact physiological mechanisms behind the exercise-induced reductions of BACE1 are unknown.

One potential mediator behind the beneficial effects of exercise is brain-derived neurotrophic factor (BDNF), as it plays a role in synaptic plasticity as well as neuronal survival and function (Patapoutian and Reichardt, 2001; Poo, 2001). Exercise training increases circulating BDNF in the periphery and BDNF content in the brain, both in human and rodent models, respectively (Rasmussen et al., 2009; Wrann et al., 2013; MacPherson, 2017; Walsh and Tschakovsky, 2018; Reycraft et al., 2019). Gold et al. (2003) demonstrated that 30 min of moderate exercise on a bicycle ergometer can significantly increase serum BDNF production in a human population, while we have demonstrated that BDNF can be exercise intensity dependent (Reycraft et al., 2019). Similar to increased circulating BDNF in human studies, BDNF mRNA and protein content is up-regulated with both chronic and acute exercise in the brains of rodent models (Soya et al., 2007; Baranowski and MacPherson, 2018). BDNF is released in response to neuronal activity and is derived from both pre- and postsynaptic sites (Waterhouse and Xu, 2009). Once released, it binds to its receptor, tyrosine receptor kinase B (TrkB), initiating downstream signaling cascades (Kaplan and Miller, 2000; Patapoutian and Reichardt, 2001). Autophosphorylation of TrkB results in the activation of several downstream intracellular targets including cAMP response element binding (CREB) and extracellular signal-regulated kinase (ERK). These targets have been linked to the neuroprotective effects of BDNF, along with mediating cellular survival, proliferation and differentiation (McAllister, 2002).

Several lines of evidence suggest that lower BDNF content could be associated with Alzheimer’s disease pathogenesis (Ferrer et al., 1999; Fumagalli et al., 2006). Clinical evidence and *in vivo* models have demonstrated that there is a reduction in expression and protein content of BDNF in Alzheimer’s disease transgenic mice and post-mortem brains of patients with Alzheimer’s disease (Phillips et al., 1991; Allen et al., 1999; Ferrer et al., 2000; Holsinger et al., 2000; Fumagalli et al., 2006; Peng et al., 2009). Similar results have been shown *in vitro* within the SH-SY5Y cell line, where administration of Aβ oligomers downregulates BDNF expression (Rosa and Fahnestock, 2015). In cortical neuron cultures, BDNF had a specific and dose-response protective effect against neuronal toxicity induced by Aβ42 (Arancibia et al., 2008). Recent work has shown that a high fat diet (HFD) leads to reduced BDNF protein content and impaired BDNF signaling that is similar to what is observed with Alzheimer’s disease (Molteni et al., 2002; Sharma et al., 2012; Morselli et al., 2014; Baranowski and MacPherson, 2018).

Despite the associations between exercise-induced increases in BDNF and reductions in BACE1 activity/content, no study has examined if there is a mechanistic link between BDNF and BACE1. This study aims to determine if BDNF has a direct effect on BACE1 and to examine the effects of an acute bout of exercise on brain BDNF content/signaling in lean and obese mice. This study further examines brain region specific differences in response to exercise and BDNF.

## Materials and Methods

### Materials

Low-fat (10% kcal fat; cat#D12450B) and high-fat (60% kcal fat; cat#D12492) diets were purchased from Research Diets Inc. (New Brunswick, NJ, United States). Horseradish peroxidase-conjugated donkey anti-rabbit and goat anti-mouse IgG secondary antibodies were from Jackson ImmunoResearch Laboratories (West Grove, PA, United States). Molecular weight marker, reagents, and nitrocellulose membranes for SDS-PAGE were acquired from Bio-Rad (Mississauga, ON, Canada) and GE Healthcare Life Science (cat#10600002). Western lightning Plus-ECL (PerkinElmer, 105001EA). Insulin and glucose were purchased from Eli Lilly and BioShop Reagents, respectively. All other sources are listed throughout the text. Antibodies against pro-BDNF (1:500, Santa Cruz cat #sc-65514), CREB (1:500, Cell Signaling cat #9197), pCREB (1:500, Cell Signaling cat#9196), TrkB (1:500, Cell Signaling cat#4603), pTrkB (1:500, Abcam cat#ab109684) APP (1:500 BioLegend, cat#SIG039152), pAPP Thr668 (1:500, Cell Signaling cat#6986S), BACE1 (1:500, Cell Signaling cat#5606P), sAPPα (1:500, BioLegend cat#SIG39139, and sAPPβ (1:500, BioLegend cat# SIG-39138) were obtained and used during this study.

### Animals

Experimental protocols were approved by the Brock University Animal Care Committee (file #17-06-02) and are in compliance with the Canadian Council on Animal Care. Male C57BL/6J mice (18 weeks of age; 31.48 ± 2.2 g, *n* = 72) were ordered from The Jackson Laboratory (Bar Harbor, ME, United States) and allowed to acclimatize for 3 days in the Brock University Comparative Biosciences Facility. During acclimatization, mice were fed standard chow (2014 Teklad global 14% protein rodent maintenance diet, Harlan Tekland, Mississauga, ON, Canada). All mice were kept on a 12-h light: 12-h dark cycle and had *ad libitum* access to food and water through the entirety of the study.

### Experimental Design

**FIGURE 1:**
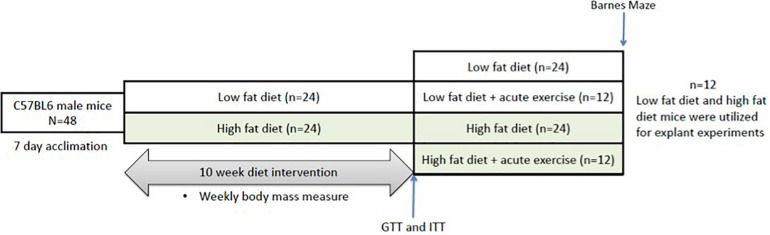
Representative image of the experimental timeline. Mice underwent 10 weeks of either low or high fat feeding followed by Barnes maze testing and GTT and ITT. GTT, glucose tolerance test; ITT, insulin tolerance test.

C57BL/6J male mice were randomized into either a HFD (*n* = 36, 60% kcal fat, cat#D12492) or low fat diet group (*n* = 36, 10% kcal fat, cat#D12450B). Baseline measures of all mice (*n* = 72) were conducted immediately after acclimatization and included body mass measurements. The mice remained on their respective diets for a 10-week period, with body mass measurements taken twice a week. Following the 10-week period, mice underwent glucose and insulin tolerance testing and Barnes maze testing. The mice were then assigned to the tissue explant study or the acute exercise study or (Figure 1). For the tissue explant study, mice were divided into two groups (Control or BDNF treated; *n* = 12 in each). For the acute exercise study, the LFD and HFD mice were randomized into either exercise or non-exercise groups [Low fat/Sedentary (LS, *n* = 24), Low fat/Exercise (LE, *n* = 12), High fat/Sedentary (HS, *n* = 24), High fat/Exercise (HE, *n* = 12)]. The exercise groups performed a single bout of exercise, 2 h of treadmill running at 15 m/min on a 5% incline as previously described (MacPherson et al., 2015; Baranowski and MacPherson, 2018). Following a 2-h recovery period, the mice were euthanized, and the prefrontal cortex and hippocampus were collected for future analysis.

### Barnes Maze Spatial Memory Test

All procedures were in accordance with Brock University’s SOP and adapted from Attar’s (Attar et al., 2013) and Rosenfeld’s studies (Rosenfeld and Ferguson, 2014). The Barnes maze test involved three phases, which were all carried out during the final week of feeding and performed at the same time each day. Visual cues were placed around the room during the habituation phase and remained in the same location for the duration of the testing phase. The habituation phase was used to acclimatize each mouse to the testing room and Barnes maze. During habituation, mice were placed under a clear container and led around the maze for 30 s. The mice were then placed over the escape cage for 3 min and gently guided in if the mouse did not enter before the time expired. Once the mouse entered, they were allowed to explore the escape cage for 1 min. The training phase was performed 24 h after the habituation phase, during which, mice were placed under a container in the center of the maze for 15 s. The container was then removed and mice were allowed to explored the maze for 2 min. If the mouse found the escape cage, they were left for 1 min before being removed. Failure to find the escape hole in 2 min resulted in guiding the mice to the escape hole using the clear container. Mice were then given a 2 min period to enter the escape cage, after which they were gently nudged in if they still did not enter. Once in the escape cage, mice were given 1 min to explore. The test phase (probe phase), was performed 48 h after the training phase. Similarly to the training phase, mice were placed in the center of the maze for 15 s under an opaque container. The container was then removed and the mice were allowed 3 min to explore and locate the escape cage. During the probe phase, latency time to the correct escape hole and permanence time spent in wrong zones were recorded (Figure 3A).

### Glucose and Insulin Tolerance Testing

Intraperitoneal glucose tolerance tests (GTT) were performed on fasted (6 h), non-anesthetized mice during the second last week of feeding (week 9). Glucose measures were obtained from tail vein blood using an automated glucometer at baseline and at 15, 30, 45, 60, 90, and 120 min following an intraperitoneal injection of glucose (2 g/kg body mass).

Intraperitoneal insulin tolerance tests (ITT) were performed on non-anesthetized mice during the second last week of feeding (week 9) following a 2-day recovery period from the GTT. Glucose measures were obtained from tail vein blood using an automated glucometer at baseline and at 15, 30, 45, 60, 90, and 120 min following an intraperitoneal injection of insulin (0.75 g/kg body mass).

### Tissue Collection

Mice were anesthetized with a weight-adjusted bolus intraperitoneal injection of sodium pentobarbital (5 mg/100 g body weight). Blood was collected, and mice were sacrificed through exsanguination. Surgical scissors were used to decapitate the mice and then a midline incision was made to remove the skin and expose the skull. Once the brain was exposed, closed curved surgical scissors were slid underneath the posterior part of the brain and gently lifted up. The scissors were then used to sever the cranial nerves, emancipating the brain. Once the brain was removed from the skull, a surgical scalpel was used to dissect the brain into the left and right hemispheres. After isolating the brain into its respective hemispheres, the prefrontal cortex and hippocampus were removed. The samples were then snap frozen in liquid nitrogen and stored at –80°C for analysis via western blotting. Inguinal white adipose tissue, epididymal white adipose tissue, and liver were also collected and used for characterization of the obesogenic model.

### Explant Experiment

The prefrontal cortex and hippocampus from the sedentary groups were dissected from the left and right hemispheres and then placed into a tube with DMEM media + 0.1% FBS and allowed to incubate/acclimate for 30 min at 37°C as described previously (Olver et al., 2018). After which, either the left or right prefrontal cortex/hippocampus were treated with 100 ng/ml of BDNF while the other side remained as a control, treated with the same volume of media and left to incubate for 30 min. The 100 ng/ml dose was selected based on previous literature utilizing similar culture models and doses (Jia et al., 2008; Robinet and Pellerin, 2010; Nigam et al., 2017) and is considered to fall within a physiological range as measured directly from CSF from the hippocampus during a behavioral task (1–10 pg/mL at baseline and 250–2,500 ng/mL at maximum) (Yoo et al., 2016). The tissue was then removed from the media, homogenized, and stored at –80° for further analysis via a BACE activity assay and Western blotting.

### Explant BACE Activity

BACE activity in the prefrontal cortex and hippocampus was determined using a commercially available beta-secretase activity assay kit (ab65357) as previously described (Yang et al., 2014; MacPherson et al., 2015). Samples were homogenized (FastPrep^®^, MP Biomedicals, Santa Ana, CA, United States) and extracted using 20 volumes of ice-cold PBS. Samples were left to incubate on ice for 15 min and centrifuged at 10,000 G for 5 min at 4°C. The supernatant was collected, and a BCA assay was performed to determine protein concentration. All samples were prepared at 0.75 μg/μL. A total of 50 μL of sample was added to each well in duplicate, followed by 50 μL of 2x reaction buffer and 2 μL of beta-secretase substrate. The plate was left to incubate in the dark at 37°C for 60 min and fluorescence was read using a spectrometer (SpectraMax M2; Molecular Devices) at excitation and emission wavelengths of 335 and 495 nm, respectively. The assay uses a secretase-specific peptide conjugated to two reporter molecules, EDANS and DABCYL. In the uncleaved form, the physical proximity of the DABCYL moiety and EDANS quenches the fluorescent signal. Cleavage of the peptide by β-secretase separates EDANS and DABCYL allowing for the release of a fluorescent signal.

### Western Blotting

Explant and post-exercise samples were homogenized (FastPrep^®^, MP Biomedicals, Santa Ana, CA, United States) in 20 volumes of NP40 Cell Lysis Buffer (Life Technologies; CAT# FNN0021) supplemented with 34 μL phenylmethylsulfonyl fluoride and 50 μL protease inhibitor cocktail (Sigma; CAT# 7626-5G, CAT# P274-1BIL). The homogenized samples were placed on a shaker in a 4°C fridge for 20 min to reduce foam accumulation. Homogenized samples were then centrifuged at 4°C for 15 min at 10,000 G, after which the supernatant was collected, and protein concentration was determined using a Bicinchoninic acid assay (Sigma-Aldrich – B9643, VWR – BDH9312). The samples were prepared to contain equal concentrations (1 μg/μl) of protein in 2x Laemmli buffer and placed in a dry bath at 100°C for 5 min. Twenty microgram of protein were loaded and separated on 10% SDS-PAGE gels for 90 min at 120 V. Proteins were then wet-transferred onto nitrocellulose membrane at 100 V for 60 min. Membranes were blocked in Tris buffered saline/0.1% Tween 20 with 5% non-fat powdered milk for 1 h at room temperature. The appropriate primary antibody (1:1000 ratio) was then applied and left to incubate on a shaker, at 4°C overnight. Following primary incubation, the membrane was washed with Tris buffered saline/0.1% Tween 20 3 × 5 min and then incubated with the corresponding secondary antibody conjugated with horseradish peroxidase (Jackson ImmunoResearch, 1:2000 ratio) for 1 h at room temperature. Signals were detected using enhanced chemiluminesence and were subsequently quantified by densitometry using a FluorChem HD imaging system (Alpha Innotech, Santa Clara, CA, United States). A representative ponceau stain was measured and analyzed for each membrane to ensure equal loading (Sander et al., 2019).

### Real-Time qPCR

To examine changes in gene expression post-exercise prefrontal cortex and hippocampus mRNA was extracted and reversed transcribed into cDNA. Changes in mRNA expression were determined using real-time quantitative PCR as described previously (MacPherson et al., 2015). RNA was isolated from the prefrontal cortex and hippocampus following homogenization in Trizol reagent using an RNeasy kit according to the manufacturer’s instructions (RNeasy Kit 74106; Qiagen). RNA yield and purity were determined using a Nano-drop system (NanoVue plus; GE healthcare). RNA samples were prepared at 1 μg/μl using RNase free water (total volume of 10 μl). cDNA was synthesized using random primers and dNTP (Invitrogen) at a 1:1 ratio as well as a master mix (5x FSB, DTT, RNase out and SuperScript II Reverse Transcriptase). 7500 Fast Real-Time PCR system (Applied Biosystems) was used to perform the RT PCR. Each sample was loaded in duplicate and contained 10 μl of PCR master mix, 4 μl of RNase free water, 1 μl of gene expression assay, and 5 μl of cDNA (diluted with 80 μl of RNase free water). Gene expression assays were purchased for *Bdnf* (Mm04230607_s1), *Bace1* (Mm00478664_m1), and *Gapdh* (Mm9999915_g1). *Gapdh* was used as a housekeeping gene and was not different between groups. Relative differences in *Bdnf* and *Bace1* mRNA expression were determined using the 2^–ΔΔ*CT*^ method and normalized to the respective control group (Livak and Schmittgen, 2001).

### Statistical Analysis

Body mass, GTTs and ITTs results were recorded, and a two-way repeated measures ANOVA analysis was performed. Effect of diet on differences on glucose and insulin tolerance area under the curve, liver, inguinal white adipose tissue, and epididymal white adipose tissue mass, and Barnes Maze spatial memory test markers were analyzed with an unpaired *t*-test. Effect of exercise within dietary groups on Barnes Maze memory testing, enzyme activity, and total and phosphorylated protein content was analyzed using a two-way ANOVA (Exercise by Diet). Significant interactions were followed up with Tukey *post hoc* analysis. For the explant experiment differences between control and BDNF treated groups were determine with a *t*-test. Data are expressed as means ± SEM with significance set at *p* < 0.05.

## Results

### Model Characterization

#### Diet-Induced Changes on Whole-Body Measurements and Cognition

**FIGURE 2:**
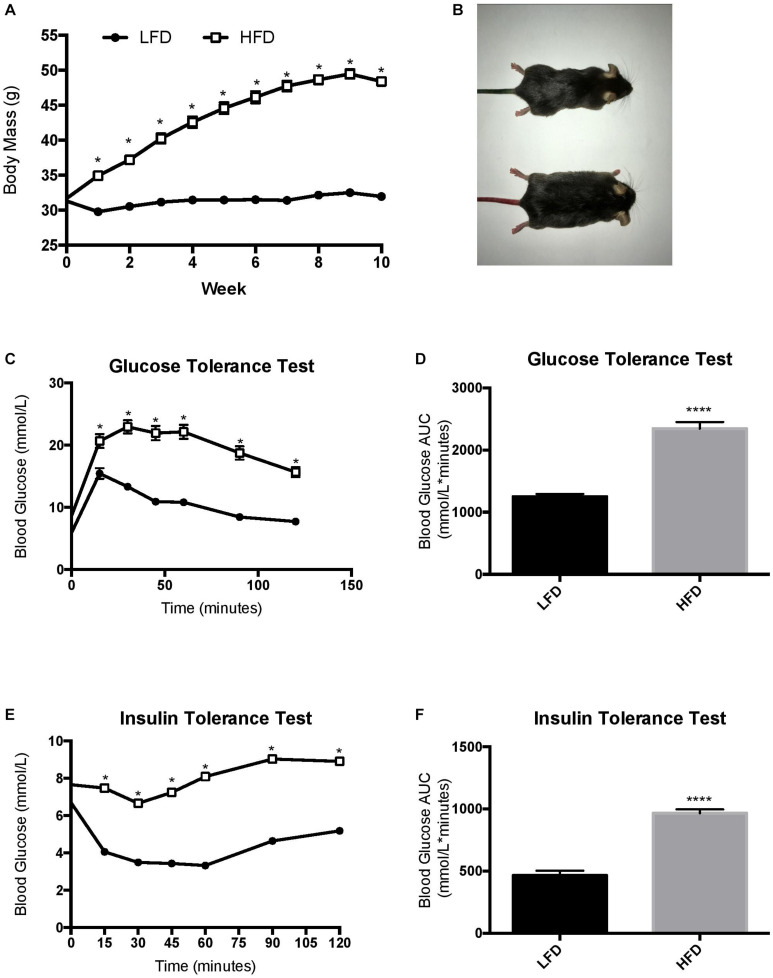
Changes in body mass throughout the duration of the study and endpoint glucose homeostasis. **(A)** LFD, animals were maintained on a low-fat diet while they aged for 10 weeks from 16 weeks of age; HFD, animals were maintained on a high fat/obesogenic diet for 10 weeks from 16 weeks of age. HFD impairs glucose and insulin tolerance. **(B)** Visual representation of mice. **(C)** Glucose tolerance test, **(D)** Glucose tolerance test represented as area under the curve, **(E)** Insulin tolerance test, **(F)** insulin tolerance test represented as area under the curve. Values are represented as mean ± SEM. Significance indicated by ^∗^ *p* < 0.05 compared to LFD. ^****^p < 0.00001 compared to LFD.

Prior to being assigned groups, there was no significant differences in body mass between the mice (*p* = 0.49). Two-way RM ANOVA testing revealed a significant interaction (*F*_10_,_700_ = 249.7; *p* < 0.0001) and main effects for diet (*F*_1_,_70_ = 256.2; *p* < 0.0001) and week (*F*_3_._387_,_237_._1_ = 360.8; *p* < 0.0001). *Post hoc* analysis demonstrated that after 1-week of the dietary intervention the HFD group had a higher body mass compared to LFD group (*p* < 0.0001). Body mass continued to be higher in the HFD group when compared to the LFD until the end of the 10-week intervention (Figures 2A,B; *p* < 0.0001). To further characterize changes in whole-body measurements, inguinal and epididymal white adipose tissue, and the liver were collected post-euthanization. The HFD groups demonstrated higher tissue mass with regards to inguinal white adipose tissue (*p* < 0.0001), epididymal white adipose tissue (*p* < 0.0001), and liver (*p* < 0.0001) when compared to the LFD groups (Table 1).

**TABLE 1:** Body and tissue mass post-10-week intervention.

	Body Mass (g)	Inguinal white adipose tissue (g)	Epididymal white adipose tissue (g)	Liver (g)
LFD	31.93 ± 0.47	0.41 ± 0.05	0.84 ± 0.07	1.20 ± 0.04
HFD	48.40 ± 0.54*	2.01 ± 0.08*	1.73 ± 0.07*	2.34 ± 0.08*

Values are presented as mean ± SEM for n = 24/group. Significance (p < 0.05) is denoted by *.

**FIGURE 3:**
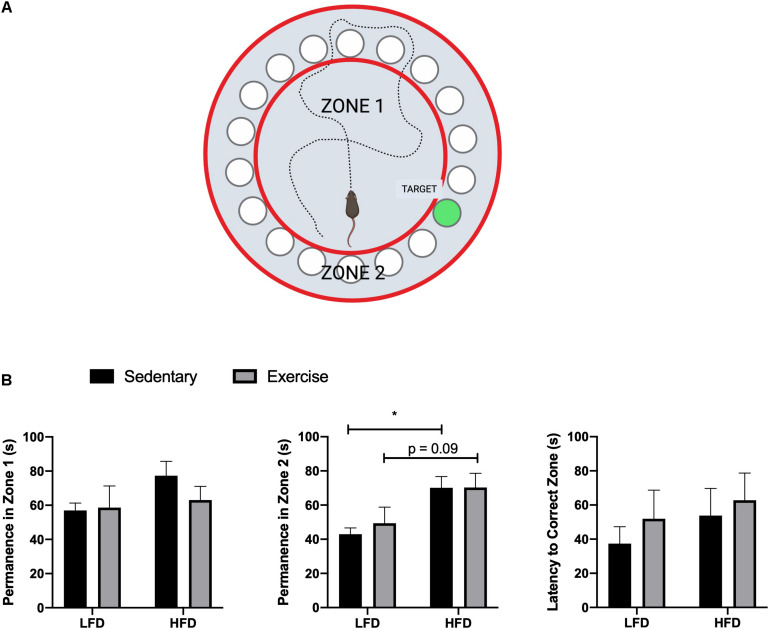
Barnes maze results. **(A)** Representative image of Barnes maze, zones, and target, created with Biorender. **(B)** Permanence time in Zone 1, Zone 2, and Latency time to the correct Zone. Values are represented as mean ± SEM. Significance indicated by * *p* < 0.05 compared to LFD.

The HFD group demonstrated a reduced whole body glucose and insulin tolerance compared to the LFD group, as indicated by a higher blood glucose at each time point after administration. Two-way RM ANOVA testing revealed a significant interaction and main effect for time and diet for the GTT (*F*_6_,_228_ = 18.77; interaction *p* < 0.0001; time *F*_3_._830_, _145_,_5_ = 112.2, *p* < 0.0001; diet *F*_1_,_38_ = 88.58, *p* < 0.0001). Two-way RM ANOVA testing revealed a significant interaction and main effect for time and diet for the ITT (interaction *F*_6_,_228_ = 15.32, *p* < 0.0001; time *F*_4_._720_,_179_._4_ = 30.05, *p* < 0.0001; diet *F*_1_, _38_ = 132.9, *p* < 0.0001) as well as a higher area under the curve in the GTT (Figures 2C,D; *p* < 0.0001) and ITT (Figures 2E,F; *p* < 0.0001). To demonstrate the detrimental effects of the HFD on cognition, a Barnes maze test was implemented to test the spatial (hippocampal) and working (prefrontal cortex) memory of the mice. Two way ANOVA analysis demonstrated a main effect of diet on greater permanence time exploring the incorrect zone (*F*_1_,_44_ = 10.72, *p* = 0.0021; Figure 3B). There was no effect of exercise (*F*_1_,_44_ = 0.197, *p* = 0.66) or interaction (*F*_1_,_44_ = 0.183, *p* = 0.67). *Post hoc* analysis demonstrated that the HFD groups had a higher permanence time compared to the LFD groups (*p* = 0.024 sedentary LFD vs. HFD; *p* = 0.09 exercise LFD vs. HFD). There were no differences in latency to the correct zone (*p* > 0.05; Figure 3B).

### Explant Study Results

#### BACE Activity Assay

**FIGURE 4:**
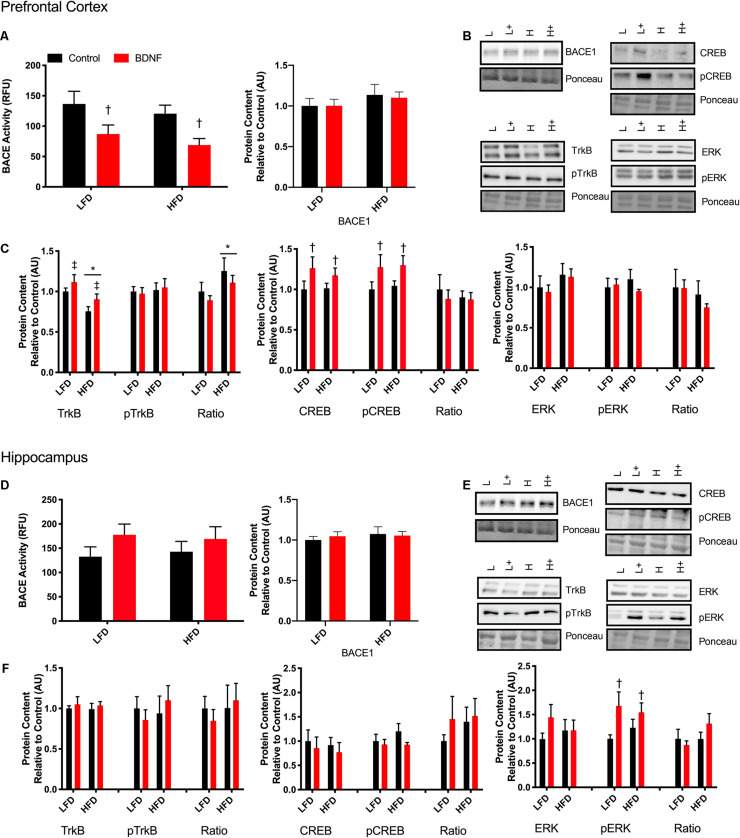
Explant study. **(A)** BDNF treatment reduces BACE activity in the prefrontal cortex from lean and obese mice, however, there were no changes in BACE1 content. **(B)** Representative immune blots for downstream markers of BDNF signaling in the prefrontal cortex. **(C)** BDNF treatment recovers HFD induced TrkB reduction and increases total and phosphorylated CREB in the prefrontal cortex. There were no detectable changes in ERK in the prefrontal cortex. **(D)** There were no detectable changes in BACE activity or content in the hippocampus with BDNF treatment. **(E)** Representative blots for downstream markers of BDNF signaling in the hippocampus. **(F)** There are no detectable changes in total or phosphorylated TrkB or CREB, however, BDNF treatment increased phosphorylated ERK content in the hippocampus. Values are represented as mean ± SEM. Significance indicated by *p < 0.05 compared to LFD, †*p* < 0.05 compared to sedentary, ‡*p* = 0.058 compared to LFD. Western blots have been cropped to provide a representative blot.

To establish if there is a direct link between BDNF and BACE1, a beta-secretase activity assay was performed on prefrontal cortex and hippocampus samples after a 30 min treatment with a 100 ng/ml dose of BDNF post-dissection. In the prefrontal cortex, there was a main effect of BDNF treatment (*F*_1_,_18_ = 9.854, *p* = 0.0057; Figure 4A). No interaction (*p* = 0.94) or diet effect (*p* = 0.30) was observed. There were no differences in BACE activity in the hippocampus samples (Figure 4D; interaction *p* = 0.68; diet *p* = 0.97; BDNF *p* = 0.12). No changes were observed in BACE1 content in either the prefrontal cortex or the hippocampus in response to the HFD or BDNF treatment (Figure 4A; interaction *p* = 0.85; diet *p* = 0.22; BDNF *p* = 0.86; Figure 4D; interaction *p* = 0.59; diet *p* = 0.51; BDNF *p* = 0.83).

### Explant Tissue Protein Content and Phosphorylation Status

To further explore the mechanisms behind the BDNF-induced reductions in BACE1 activity, Western blotting was performed on downstream targets of BDNF. In the prefrontal cortex, two way ANOVA analysis demonstrated a main effect for diet on TrkB content (*F*_1_,_41_ = 12; *p* = 0.0012) and a main effect for BDNF treatment (*F*_1_,_41_ = 4.054; *p* = 0.051; Figures 4B,C). There was no interaction (*F*_1_,_41_ = 0.056; *p* = 0.81). There was no effect of diet or BDNF treatment on phosphorylated TrkB. Analysis of the ratio of phosphorylated to total TrkB content demonstrated a main effect of diet (*F*_1_,_38_ = 4.53; *p* = 0.039). There was a main effect of BDNF treatment on total CREB content (*F*_1_,_41_ = 4.26; *p* = 0.045; Figure 4C) and on phosphorylated CREB content (*F*_1_,_41_ = 5.60; *p* = 0.025; Figure 4C). In the prefrontal cortex, there were no differences in total or phosphorylated ERK with BDNF treatment (*p* > 0.05; Figure 4C).

In the hippocampus, there were no changes in total or phosphorylated TrkB content, total or phosphorylated CREB content (*p* > 0.05; Figures 4E,F). Two way ANOVA analysis demonstrated that there was a main effect for BDNF treatment to increase phosphorylated ERK in the hippocampus regardless of diet (*F*_1_,_39_. = 6.357; *p* = 0.017; Figure 4F). There were no differences in total ERK content (*p* > 0.05; Figure 4F).

### Exercise Study Results

#### BDNF Signaling

**FIGURE 5:**
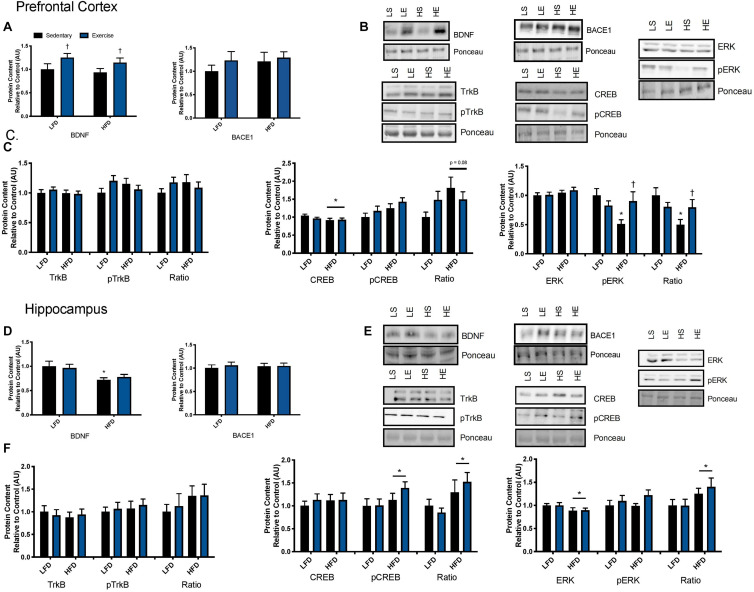
Exercise induced BDNF signaling. **(A)** An acute bout of exercise increased BDNF total protein content in the prefrontal cortex, however, there were no changes in BACE1 content. **(B)** Representative blots for downstream markers of BDNF signaling in the prefrontal cortex. **(C)** There were no effects of diet or acute exercise on TrkB, total CREB content was reduced with a HFD, and phosphorylated ERK was significantly lower with a HFD, however, phosphorylated ERK was recovered with a single bout of exercise, represented as an absolute value and relative to total content in the prefrontal cortex. **(D)** BDNF content was lower with the HFD in the hippocampus and no effect of exercise, there were no changes in BACE1 content. **(E)** Representative blots for downstream markers of BDNF signaling in the hippocampus. **(F)** There were no effects of diet or acute exercise on TrkB, CREB phosphorylation was higher with a HFD represented as both an absolute value and as a ratio to total content, and total ERK was reduced with a HFD and the ratio of pERK/ERK was significantly higher in the HFD group in hippocampus. Values are represented as mean ± SEM. Significance indicated by * *p* < 0.05 compared to LFD, †*p* < 0.05 compared to sedentary. Western blots have been cropped to provide a representative blot.

To examine the effect of acute exercise on BDNF and BDNF signaling, LFD and HFD mice underwent an acute bout of treadmill exercise. BDNF content in the prefrontal cortex was not altered with the HFD. Acute exercise resulted in a main effect for increased BDNF content in the prefrontal cortex regardless of dietary intervention (Figures 5A,B; *p* = 0.02). In the hippocampus, there was a main effect of diet on BDNF content in the hippocampus (Figures 5D,E; *p* = 0.003). *Post hoc* analysis demonstrated that BDNF content was lower in the HFD group compared to the LFD group (*p* = 0.05). No changes were observed in BACE1 content in either the prefrontal cortex or the hippocampus in response to the HFD or acute exercise (*p* < 0.05, Figures 5A,D).

To determine if these changes in BDNF content resulted in changes to downstream signaling the BDNF receptor, TrkB, was examined as well as downstream signaling markers CREB and ERK. No changes were observed in total or phosphorylated content of TrkB in either the prefrontal cortex or the hippocampus in response to the HFD or acute exercise (*p* < 0.05, Figures 5C,F. In the prefrontal cortex, there was no interaction or main effect of exercise, there was a main effect of diet where the HFD group had lower total CREB content (*F*_1_,_43_ = 3.17; *p* = 0.03; Figure 5C). There were no changes in CREB phosphorylation or in the ratio of phosphorylated to total content (*p* > 0.05). In the hippocampus, there were no changes in CREB total protein content. There was a main effect of diet on CREB phosphorylation where the HFD group had higher CREB phosphorylation (*F*_1_,_43_ = 3.00; *p* = 0.03; Figure 5F). This main effect of diet was also observed in the ratio of phosphorylation to total content (*F*_1_,_43_ = 5.88; *p* = 0.019; Figure 5F).

In the prefrontal cortex, there were no changes in total ERK content (*p* > 0.05; Figure 5C). Two way ANOVA analysis demonstrated an interaction effect (*F*_1_,_44_ = 6.15; *p* = 0.017) and *post hoc* analysis revealed that the sedentary HFD group had lower ERK phosphorylation compared to the sedentary LFD group (*p* = 0.001) while a single bout of exercise increased ERK phosphorylation in the HFD group (*p* = 0.01). This interaction effect was also demonstrated with the ratio of phosphorylated to total ERK (*F*_1_,_44_ = 5.00; *p* = 0.03; Figure 5C) and *post hoc* analysis revealed that the HFD group had lower ERK phosphorylation within the sedentary groups (*p* = 0.015) while a single bout of exercise increased ERK phosphorylation in the HFD group (*p* = 0.001). In the hippocampus, there was a main effect of diet where the HFD group had lower ERK content (*F*_1_,_43_ = 4.3; *p* = 0.045; Figure 5F). There were no differences in phosphorylated ERK (*p* > 0.05; Figure 5F). There was a main effect for an increase in the ratio of phosphorylated to total protein ERK in the HFD, likely due to the reduction of total ERK (*F*_1_,_41_ = 5.11; *p* = 0.029).

#### mRNA Expression

**FIGURE 6:**
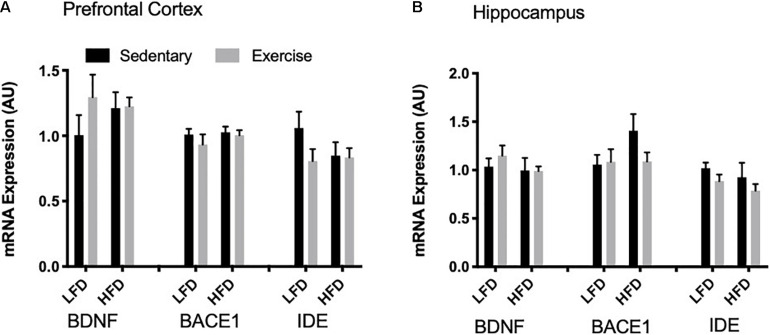
mRNA expression. **(A)** There were no detectable changes in BDNF, BACE1 and IDE mRNA expression in the prefrontal cortex. **(B)** There were no detectable changes in BDNF, BACE1 and IDE mRNA expression in the hippocampus. Values are represented as mean ± SEM.

To detect acute changes with the single bout of exercise and the long-term effect of a HFD, mRNA expression of *Bdnf*, *Bace1*, and *Ide* were measured via RT-qPCR. In the prefrontal cortex and the hippocampus there were no changes in *Bdnf* mRNA with exercise or the HFD (*p* > 0.05; Figures 6A,B). Similarly, there were no changes in *Bace1* mRNA expression in both the prefrontal cortex and hippocampus with exercise or HFD. In the prefrontal cortex and hippocampus, there were no detectable changes in mRNA expression of *Ide* (*p* > 0.05).

## Discussion

Results from this study demonstrate a novel link between BDNF and BACE1 activity. Direct treatment of brain explants with BDNF resulted in a region specific decline in BACE1 activity in the PFC but not the hippocampus. Further, this study provides a comprehensive characterization of the effect of HFD and an acute bout of exercise on brain BDNF content and signaling in a non-transgenic, preclinical model of sporadic Alzheimer’s disease. To the best of our knowledge, this is the first study to demonstrate a direct effect of BDNF treatment on BACE1 activity and provides further novel insight into the potential mechanisms responsible for previously shown exercise-induced reductions in BACE1 activity (MacPherson et al., 2015).

### Treatment With BDNF Reduces BACE1 Activity

By directly treating prefrontal cortex and hippocampal explants we uncovered two novel aspects of the role of BDNF in the brain. First, we demonstrated that treatment with BDNF reduced BACE1 activity in the prefrontal cortex but not in the hippocampus – highlighting the second novel aspect of regional differences in the response to BDNF. This novel finding demonstrates the effect of BDNF on reducing BACE1 activity, independent of other alterations that may arise due to exercise and that there is a direct link between BDNF signaling and BACE1 regulation. In our hands, BDNF treatment did not alter BACE1 total protein content, suggesting that BDNF modulates BACE1 activity through a mechanism independent of gene transcription and translation, or alterations in protein degradation. Being the rate-limiting enzyme in the amyloidogenic pathway and playing a large role in the production of the neurotoxic intermediate, Aβ, aberrant BACE1 activity is considered pivotal to Alzheimer’s disease progression (Hardy, 1992; Hardy and Higgins, 1992; Selkoe, 2004; Gandy, 2005; Hampel and Shen, 2009). Due to the normal physiological roles of BACE1 in the brain, complete inhibition or knockout of BACE1 results in fatal phenotypes and outcomes (Vassar, 2014), such as, increased BACE1 protein levels (Liu et al., 2019), worsened cognition and daily function (Egan et al., 2019), increased axon targeting errors (Rajapaksha et al., 2011), reduced myelination, endophenotypes of schizophrenia (Savonenko et al., 2008), and seizures (Hitt et al., 2010). Thus an approach, such as exercise, to modulate BACE1 activity, rather than inhibiting it altogether, is crucial.

Surprisingly, there were no changes in BACE activity with BDNF treatment in the hippocampus, although this compliments the unresponsiveness of BDNF signaling to an exercise stimulus seen within the exercise study. Preliminary work in our lab has shown that TrkB total protein content is significantly lower in the hippocampus when compared to the prefrontal cortex (data not shown; *p* = 0.008). The reduced content of TrkB in the hippocampus may have influenced the responsiveness to BDNF treatment and explain the lack of response to exercise. A recent time course study from our lab demonstrated that an acute bout of exercise was able to reduce BACE1 content in the prefrontal cortex after a 2-h recovery period; whereas, the hippocampus required an 8-h recovery period before a reduction in BACE1 content was observed (Yang A. J. et al., 2019). It is possible that the hippocampus needed either a longer intervention, incubation time, or recovery time to elicit a response; however, because the mechanisms that link BDNF to BACE1 remain elusive, it is difficult to explain this difference in brain region response.

An examination of markers related to BDNF signaling provided some insight into the potential mechanism linking BDNF to reduced BACE1 activity. In the prefrontal cortex, the region where BACE1 activity was reduced with BDNF treatment, we observed elevated levels of TrkB content (*p* = 0.058) despite initial impairments by the HFD. Despite observing elevated levels of TrkB content, there were no changes in TrkB phosphorylation. Although the exact reason for this remains unknown, it can be speculated that this is due to a discrepancy between the time point of treatment to the point of tissue collection. The turnover rate of TrkB autophosphorylation remains to be determined and was potentially missed in this study. Additionally, BDNF treatment resulted in significant increases in CREB and pCREB content; however, there were no changes in BDNF’s downstream effecter ERK. This suggests that BDNF downstream signaling through CREB potentially plays a role in BACE1 modulation. In the hippocampus there were no changes in BDNF signaling through TrkB or CREB, however, there was a significant increase in pERK. Despite ERK being downstream of TrkB signaling, ERK has been shown to be activated by BDNF via other mechanisms (Easton et al., 1999), thus potentially explaining the increase in pERK seen with BDNF treatment in the hippocampus. These results may help explain why BDNF treatment did not reduce BACE1 activity in the hippocampus. Future work will be required to fully dissect these relationships.

### Effects of a High Fat Diet and Acute Exercise on Brain BDNF

The ability to develop reliable models that can replicate late-onset sporadic AD is an immense hurdle in identifying and targeting the causative factors of the disease. In an effort to accomplish this, our group has demonstrated a link between diet-induced obesity and the early preclinical pathogenesis of AD in C57BL6/J models (MacPherson et al., 2015; Baranowski and MacPherson, 2018; Baranowski et al., 2018a; Yang A. J. et al., 2019; Yang A. J. T. et al., 2019). Here we further characterize the influence of an obesogenic diet on BDNF content and signaling as well as the effects of an acute bout of exercise on BDNF content and signaling. In our model, we demonstrated that chronic high fat feeding resulted in whole body glucose intolerance and insulin resistance as well as impairments in cognitive ability/spatial memory in the Barnes maze. This is in agreement with previous literature showing that a HFD can lead to insulin resistance and is linked to the progression of Alzheimer’s disease (Kern et al., 2001; de la Monte and Wands, 2008; Bigornia et al., 2012; Candeias et al., 2012; Duarte et al., 2012; Vagelatos and Eslick, 2013), as well as HFD-induced impairments to cognitive ability and memory (Thirumangalakudi et al., 2008; Maesako et al., 2012; Vandal et al., 2014). The current study demonstrates that HFD-induced impairments in BDNF content and signaling, specifically we saw reductions in total BDNF content, its receptor TrkB, and downstream targets CREB and ERK. Growing evidence suggest that decreases in BDNF content and defects in its signaling pathway via its receptor, TrkB, are associated with neurodegeneration and the progression of Alzheimer’s disease (MacPherson, 2017). Collectively, these findings suggest that the consequences of HFD-induced reductions in BDNF could be early contributors to Alzheimer’s disease progression.

Previous work in our lab has shown that an acute bout of exercise significantly reduced BACE1 activity in the prefrontal cortex in a C57BL/6 obesogenic mouse model (MacPherson et al., 2015); however the mechanism behind this were elusive. In the current study using a similar model, we have demonstrated that a single bout of exercise results in higher levels of BDNF protein content in both LFD and HFD groups in the prefrontal cortex. This increase in BDNF protein content was not accompanied by an increase in mRNA at the same time point. This is not entirely unexpected, as typically increases in BDNF protein content follows increases in *Bdnf* mRNA and we may have missed the timepoint at which mRNA peaked. Few studies have examined a full time course of changes in BDNF expression and protein content post- an acute bout of exercise. Rasmussen et al. (2009) did observe increased *Bdnf* mRNA 2 and 6 h post-exercise in the PFC and hippocampus, however they did not measure BDNF protein content and the acute exercise protocol utilized was different from the one in the current study. If an understanding of the exercise induced time course of changes in BDNF is of interest the future studies may want to focus on both mRNA and protein content changes at several time points post-exercise. Using exercise to increase BDNF content is heavily supported in the literature (Cotman et al., 2007; Rasmussen et al., 2009; Wrann et al., 2013; Hamilton and Rhodes, 2015; MacPherson, 2017; Baranowski and MacPherson, 2018; Walsh and Tschakovsky, 2018); however, it was important to validate this in our model and to show that BDNF signaling was still recoverable despite the detriments from chronic high fat feeding. Our work not only highlights that an acute bout of exercise can increase BDNF content but also that it can recover BDNF downstream signaling, ERK phosphorylation. ERK has a role in synaptic plasticity, memory, and is thought to facilitate these effects via the phosphorylation of CREB (Patapoutian and Reichardt, 2001). ERK plays a pivotal role in BDNF signaling and eliciting its neuroprotective effects, thus it is promising to show that an acute bout of exercise is able to recover ERK phosphorylation in the prefrontal cortex.

Our study highlights that: (1) BDNF can directly reduce BACE1 activity, and (2) a single exercise bout elevates brain BDNF content. Choi et al. (2018) found that by combining the promotion of neurogenesis and elevating BDNF levels in 5x FAD mice (a familial model of Alzheimer’s disease), they were able to mimic the beneficial effects of exercise with regards to the improvement of cognitive ability and spatial memory. Our findings exhibit comparable implications, specifically in a pre-clinical sporadic model of Alzheimers disease, while also elucidating a potential mechanism behind the neuroprotective effects of BDNF. These results in conjunction with our findings that direct BDNF treatment mimics exercise-induced reductions in BACE1 activity, suggests that BDNF could be a player behind the therapeutic effects of exercise on BACE1 regulation and impaired cognition, however, further work is needed to corroborate this. Furthermore, these findings indicate that BDNF may be a promising avenue in the development of drug therapies in the prevention or treatment Alzheimer’s disease.

### Limitations

The current data provides novel insight into establishing a link between BDNF and BACE1 activity, however, this work lacks the confirmation that exercise-induced increases in BDNF content can lead to exercise-induced reductions in BACE1 activity. Future research is needed to identify whether BDNF is required for exercise-induced reductions in BACE1 activity. Further, although we utilized a physiologically relevant dose of BDNF in the explant studies, if these concentrations of BDNF are elicited in the brain with an exercise bout remains to be determined.

### Perspectives

This study provided novel information regarding the mechanistic link between BDNF and BACE1 regulation and demonstrated high fat feeding-induced impairments to BDNF signaling, cognition, and glucose homeostasis. Furthermore, this study highlights that BDNF levels are elevated with a single bout of exercise in an obesogenic model and that exercise can recover HFD-induced impairments to BDNF signaling. The exercise study demonstrated exercise-induced increases in BDNF within the same model that showed exercise-induced reductions in BACE1 activity (MacPherson et al., 2015). More interestingly, the explant study is the first to elucidate a direct link between BDNF and BACE1. We have shown that with BDNF treatment, in the absence of exercise, BACE1 activity is significantly reduced. The information provided by our study contributes to our understanding of the underlying mechanisms behind APP processing and more specifically behind modulating BACE1 activity. This is valuable as it will allow the possibility of designing and developing evidence-based preventative or therapeutic interventions, both by using exercise programs and pharmacological approaches, for populations that cannot exercise or are at a greater risk of Alzheimer’s disease.

Here we demonstrate that BDNF can reduce BACE1 activity, however the exact mechanism(s) behind this interaction remains elusive. Understanding how BACE1 is regulated may provide future insight into how BDNF modifies the amyloidogenic cascade. BACE1 can be regulated through various post-translational modifications including: palmitoylation, acetylation, as well, through phosphorylation (Araki, 2016). Of particular interest, BACE1 can be phosphorylated at the Thr252 site (Song et al., 2015). Song et al. (2015) demonstrated that phosphorylation of BACE1 increased its activity *in vitro*, whereas phosphorylation-defective mutant BACE1 showed lower activity and Aβ production compared to wild-type BACE1. Future research should explore whether BDNF influences the phosphorylation of BACE1 at the Thr252 site. Elucidating the mechanisms underlying the connection between BDNF and BACE1 is crucial as it will further our knowledge on BDNF’s neuroprotective effect and its role in BACE1 regulation and allow the development of future therapeutic interventions.

## Data Availability Statement

The raw data supporting the conclusions of this article will be made available upon request by the authors, without undue reservation.

## Ethics Statement

The animal study was reviewed and approved by Brock University Animal Care Committee (file #17-06-02) and are in compliance with the Canadian Council on Animal Care.

## Author Contributions

BB: conceptualization, investigation, methodology, formal analysis, data curation, visualization, writing – original draft, and writing – review and editing. GH and DM: investigation, methodology, data curation, and writing – review and editing. RM: conceptualization, formal analysis, visualization, project administration, funding acquisition, supervision, writing – original draft, and writing – review and editing. All authors contributed to the article and approved the submitted version.

## Conflict of Interest

The authors declare that the research was conducted in the absence of any commercial or financial relationships that could be construed as a potential conflict of interest.
